# How Strongly Connected Are Positive Affect and Physical Exercise? Results From a Large General Population Study of Young Adults

**DOI:** 10.32872/cpe.v2i4.3103

**Published:** 2020-12-23

**Authors:** Sarah D. Pressman, Keith J. Petrie, Børge Sivertsen

**Affiliations:** a Department of Psychological Science, University of California Irvine, Irvine, CA, USA; b Department of Psychological Medicine, University of Auckland, Auckland, New Zealand; c Department of Health Promotion, Norwegian Institute of Public Health, Bergen, Norway; d Department of Research & Innovation, Helse-Fonna HF, Haugesund, Norway; e Department of Mental Health, Norwegian University of Science and Technology, Trondheim, Norway; University of Leuven, Leuven, Belgium

**Keywords:** positive affect, exercise, physical exercise, vigor, well-being

## Abstract

**Background:**

Previous research has shown a link between low positive affect (PA) and numerous physical and psychological well-being outcomes but, recent research has raised the possibility that this relationship may be driven by physical activity. Thus, we were interested in exploring the PA-exercise connection by examining this relationship across differing levels of body mass and athleticism. We also looked at whether the item “active” that is used in many PA assessments was responsible for this effect.

**Method:**

Participants were part of the Norwegian SHoT2018 national survey of 50,054 young adults (mean age = 23.2, 68.9% women), who completed electronic surveys about their exercise levels (duration, frequency and intensity) and affect.

**Results:**

There was a clear and strong dose-response association between current state PA and the duration, frequency and intensity of exercise. For example, duration, magnitude, and slope effects were strongly driven by regular exercisers who had more than a 20-fold greater likelihood of being in the highest PA deciles compared to the least frequent exercisers. These dose-response connections replicated across both healthy and overweight BMIs, as well as in elite athletes. Removing the word “active” from the PA measure substantially reduced the size of this association, although the dose-response relationship remained.

**Conclusion:**

The observed strong connections have critical implications for health researchers and clinicians, and point to a need to carefully consider what types of activities are most strongly tied to well-being.

## Abstract (highlights)

- There is a strong dose-response connection between exercise and positive emotion.
- This pattern persists across a range of body types and athlete characteristics.
- Overlap between adjectives in affect and self-reported activity scales partially but not fully explains the connection.
- Past work showing benefits of happiness on health may be partially or primarily driven by activity, not emotion.

It has long been established in both research and in common public knowledge that exercise can lead to greater positive affect (PA; Arent, Landers, & Etnier, 2000; Elavsky et al., 2005) as well as a reduction in negative affect (NA; e.g., depression) (Berger & Owen, 1983; Ströhle, 2009). While perhaps less recognised, it is also true that people high in PA engage in more physical exercise, as well as other positive health behaviors (Boehm et al., 2018; Cohen & Pressman, 2006) indicating potentially bidirectional and strongly interconnected associations between these two variables (Pasco et al., 2011). Recent work even indicates the value of positive psychology interventions for increasing physical exercise in the context of illness and stress (Huffman, Millstein, et al., 2020). With the common goal of improving well-being in patient samples, it is critical that we more fully explore this question so as to better inform the value of positive psychology interventions in clinical populations.

Understanding the nature of this association becomes even more critical given the burgeoning literature connecting PA to better physical health across a wide range of domains (Chida & Steptoe, 2008; Diener & Chan, 2011; Pressman, Jenkins, & Moskowitz, 2019). This includes longitudinal studies showing that PA predicts later health outcomes such as longevity (Danner, Snowdon, & Friesen, 2001; Pressman & Cohen, 2012; Willroth, Ong, Graham, & Mroczek, 2020), infectious illness (Cohen, Alper, Doyle, Treanor, & Turner, 2006; Cohen, Doyle, Turner, Alper, & Skoner, 2003), heart disease (Boehm & Kubzansky, 2012), HIV severity (Moskowitz, 2003) and other morbidities, even after accounting for critical covariates such as baseline health, medication use, negative affect, and other relevant factors. These types of studies, as well as recent positive psychology interventions showing improvements in later self-reported health (Kushlev et al., 2020) and mental health outcomes in diseased samples (see review by Pressman, Jenkins, & Moskowitz, 2019) point to the interesting possibility that PA can *cause* better health.

This work has helped foster a new field of “Positive Health” research (Seligman, 2008) as well as a burgeoning area of research trying to improve health via Positive Psychology interventions (e.g., Huffman et al., 2011; Moskowitz et al., 2017, 2012). However, as discussed recently (Pressman & Cross, 2018), this literature becomes potentially less compelling and the focus on positive psychology interventions for health promotion less useful, if the reason that the PA-health association is largely due to the overlap between positive affect and physical exercise or fitness. That is, is PA correlated with better health primarily because happy people are also more physically active, and therefore *healthier* people? Recent evidence confirming the causal effects of positive psychological interventions on increased exercise and general activity points to this possibility (e.g., Huffman, Feig, et al., 2019).

This problem is compounded by the fact that when utilizing self-report scales, there can be a large overlap between physical health self-reports and PA self-reports. For example, many popular affect measures rely on adjectives like “active” and “energetic” to tap positive affect (McNair, Lorr, & Droppleman, 1971; Watson, Clark, & Tellegen, 1988). While these items do tap feelings of vitality important to the conceptualization of PA, critically, they also tap physical fitness and perceived health, as evidenced by frequently used self-reported health scales that use these types of items (Kind, Brooks, & Rabin, 2005; McNair et al., 1971). That is, if we take the word “active” (an item from the Positive and Negative Affect Schedule [PANAS]; Watson, Clark, & Tellegen, 1988) literally, then someone *feeling* active may also *be* more (physically) active. Assessments do not distinguish between psychological versus physical forms of these vigorous feelings. This is problematic because to the extent that these measures represent the same underlying construct, it may be that feelings of happiness and joy are not predicting future health, but rather that it is health predicting health.

We examined this issue recently in a large sample of over 5000 older adults (Petrie et al., 2018). Consistent with past PA-mortality research (Chida, Hamer, Wardle, & Steptoe, 2008), lower PA was associated with nearly *double* the mortality risk over a 16-year follow-up as compared to those with the highest PA. However, when unpacking the subtypes of PA responsible for this effect, we found that the association was primarily driven by the active item of the PANAS. This effect remained after accounting for the effects of the remaining PANAS items, demographics, and other important covariates. Thus, it was not the more emotionally laden and less activity/arousal based items driving longevity but the PANAS activity item. While we did control for exercise in analyses as well, a limitation was that physical activity was assessed by only a single 3-point item asking about weekly level of exercise which did not allow us to look more closely at the nature of the PA (or felt activity) and exercise connection.

This minimalist approach to assessing physical activity is echoed across the PA-health literature, including in studies showing that it is the high and not low energy components of PA most tied to reduced mortality (no activity control) (Pressman & Cohen, 2012) and decreased susceptibility to catching the common cold (included a simple measure of days exercised multiplied by effort) (Cohen et al., 2003). This practice is also common in studies on the connections between general PA and longevity where studies use single yes/no item regarding vigorous activity (Steptoe & Wardle, 2011) or no activity assessment (Danner, Snowdon, & Friesen, 2001). Unfortunately, it is also the case that many studies focused on physical activity do minimal assessments of PA, relying, for example on assessments of only one type of PA (e.g., vigor) or instead infer PA and well-being because of a drop in mental health problems like depression or anxiety symptoms (Berger & Motl, 2000; Penedo & Dahn, 2005; Schinke, Stambulova, Si, & Moore, 2017).

Thus, clearly there is a need to examine the association between these related variables in more detail where PA can be compared to a range of activity markers across a large number of individuals. Furthermore, given the concern about high PA simply being a marker of healthy fitness, this should be tested in those both high and low in fitness. This will enable a deeper understanding of the degree of connection association between fitness and high arousal PA, and more clarity about past research linking PA to health and mortality. To examine the extent to which PA and physical activity are overlapping constructs, we used data from the SHoT2018 study, a sample of over 50,000 Norwegian young adults. We hypothesized that PA would be strongly associated with *all* measures of self-reported physical exercise including exercise frequency, intensity and duration. In addition, we consider several previously unexplored avenues. We capitalized on the survey questions that distinguished young people who self-identified as elite athletes. This allowed us to examine whether top athletes had significantly greater odds of also having higher PA, as well as whether the opposite would be true in individuals with high Body Mass Index. The large size of the sample also enabled an examination of whether the PA-physical exercise connections holds between men and women and within each of the frequency, intensity and duration of exercise dimensions. Finally, based on its importance in our past work, we explored to what extent the associations found in the above analyses changed when the word “active” was removed from the PANAS PA measure and the size of the association of feeling “active” with these exercise measures.

## Method

### Participants

The SHoT study (an acronym for the Norwegian name: Studentenes Helse- og Trivselsundersøkelse *[Students’ Health and Wellbeing Study]*) is a national student survey for higher education in Norway. Details of the study have been published elsewhere (Sivertsen, Råkil, Munkvik, & Lønning, 2019). So far, three health surveys of the student population in Norway have been completed (2010, 2014, and 2018). Both the size and scope of the SHoT studies have expanded over time, and now include detailed information on both mental and physical health, quality of life, and health-related behaviours.

The SHoT2018 study was a joint effort between the three largest student welfare organizations in Norway and the Norwegian Institute of Public Health (NIPH). The study was conducted between February 6^th^ and April 5^th^, 2018, on all full-time Norwegian students taking higher education (both in Norway and abroad). The collection of the health survey was in close collaboration with all the student welfare organizations in Norway. Students were told that participation was completely voluntary, and that there were no penalties for not filling out the survey. Eight percent of the sample were immigrants, defined as either the student or his/her parents being born outside of Norway.

The study protocol was approved by the Regional Committee for Medical and Health Research Ethics of Western Norway (no. 2017/1176/REK vest), whose directives are based on the Declaration of Helsinki. Written electronic consent was obtained from all subjects included in this study.

### Measures

#### The Positive and Negative Affect Schedule (PANAS)

The PANAS (Watson et al., 1988) is a 20-item questionnaire which comprises two subscales, one that measures positive affect (PA) and the other which measures negative affect (NA). The PA scale of interest here includes the terms interested, alert, enthusiastic, excited, proud, inspired, strong, active, and attentive. Participants are instructed to rate to what extent they experience each emotion right now, rated on a 5-point scale from “very slightly or not at all” (coded as 1) to “extremely” (coded as 5). A sum score is calculated with higher scores representing greater PA. For the purpose of the present study, the sum scores were divided into both tertiles and deciles separately for men and women. The Cronbach’s alpha for the PA subscale in the current study was 0.91. The NA subscale was not included in the SHoT study^1^1NA was not the focus of the paper given our past work showing that it does not alter PA-health associations (Petrie et al., 2018), extensive work on the independence of PA and NA in the PANAS (Watson et al., 1988), existing work on this sample examining NA and mental health (Grasdalsmoen, Eriksen, Lønning, & Sivertsen, 2020), and the fact that the questions here target the potential overlap specific to PANAS PA (not NA) and physical activity measurements..

#### Physical Exercise

The students were first presented with the following brief definition of physical exercise: “With physical exercise, we mean that you, for example, go for a walk, go skiing, swim or take part in a sport.” Physical exercise was assessed using three sets of questions, assessing the average number of times exercising each week, and the average intensity and average hours each time: 1) *“How frequently do you exercise?”* (Never, Less than once a week, Once a week, 2–3 times per week, Almost every day); 2) *“If you do such exercise as frequently as once or more times a week: How hard do you push yourself?* (I take it easy without breaking into a sweat or losing my breath, I push myself so hard that I lose my breath and break into a sweat, I push myself to near-exhaustion); and 3) *“How long does each session last?”* (Less than 15 minutes, 15–29 minutes, 30 minutes to 1 hour, More than 1 hour”. Detailed results of college students’ exercise in the SHoT studies have been published elsewhere (Grasdalsmoen, Eriksen, Lønning, & Sivertsen, 2019). This 3-item questionnaire has previously been used in the large population-based Nord-Trøndelag Health Study (the HUNT studies). Previous validation studies (Kurtze, Rangul, Hustvedt, & Flanders, 2008) have demonstrated moderate-to-strong correlations between the questionnaire responses, and direct measurement of VO_2_max (*r* = 0.48), (an objective indicator of cardiorespiratory fitness) during maximal work on a treadmill, with ActiReg (*r* = 0.39), an instrument that measures PA and energy expenditure (EE), and with the International Physical Activity Questionnaire (IPAQ; *r* = 0.55). Respondents were also asked if they considered themselves to be a “top athlete” (yes/no), and if so, how many hours per week they trained (drop-down menu: 0 to 40 hours).

### Statistical Analyses

IBM SPSS Statistics 25 for Mac (SPSS Inc., Chicago, IL) was used for all analyses. Multinomial logistic regression models were computed to assess the association between levels of physical exercise (independent variable; lowest level of the three physical exercise variables being the reference category) and deciles of PA (dependent variable; first decile being the reference category). Being similar to binary logistic regression, multinomial regression is used when the dependent variable is nominal with more than two levels. Results are presented as odds-ratios (ORs) with 95% confidence intervals (95% CIs). There was very little missing data on the PA items, with missing responses ranging from *n* = 167 (1.1%) to *n* = 1092 (2.6%), and hence techniques involving multiple imputations were not considered, and missing values were handled using listwise deletion.

## Results

### Descriptive Statistics

In terms of *frequency* of physical exercise, 24% of the sample reported being physically active “every day”, while 47% responded exercising “2-3 times per week”. Moreover, 16%, 12% and 4% reported training “once a week”, “less than once a week”, or “never”, respectively. Regarding the students’ reports of their average physical exercise *intensity*, 11% of the sample responded “I push myself to near-exhaustion”, while 71% reported “I push myself so hard that I lose my breath and break into a sweat” and 18% responded “I take it easy without breaking into a sweat or losing my breath”. On the item assessing the *duration* of each episode of physical exercise, 37% reported an average duration of “more than 1 hour”, compared to 52%, 10% and 2% reporting “30 minutes to 1 hour”, “15–29 minutes”, and “less than 15 minutes”, respectively.

**Figure 1:**
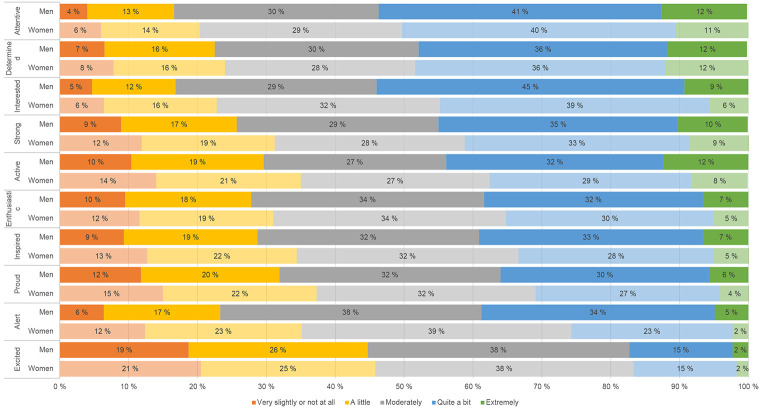
Distribution of Positive Affect Items in Men and Women in the SHoT2018 Study *Note.* Sorted by proportion of students reporting each item “quite a bit” or “extremely”.

The response distribution of the 10 PA items for both men and women are presented in Figure 1. As shown, the proportion of students responding feeling “attentive” either quite a bit or extremely was 51%, followed by “determined” (48%) and “interested” (47%). In contrast, only 17% of the sample responded feeling “excited” quite a bit or extremely. There were only marginal sex differences in terms of the response distribution of PA items.

### Is Positive Affect Associated With Physical Exercise?

The physical exercise characteristics according to sex-specific tertiles on the PA-scale are presented in Table 1. Low PA scores were more prevalent among those with lower exercise levels. These trends were present in a dose-response manner, and evident across all four physical exercise items (see Table 1 for details).

**Table 1:** Age Group and Physical Exercise Characteristics by Positive Affect (PA) Tertile Stratified by Sex in the SHoT2018 Study, Norway, 2018

Group Characteristics	Women			Men		
	PA Lower tertile	PA Middle tertile	PA Upper tertile	PA Lower tertile	PA Middle tertile	PA Upper tertile
Age group						
18-20 years	36.0%	34.8%	29.2%	36.5%	32.8%	30.7%
21-22 years	33.5%	34.7%	31.8%	31.7%	34.0%	34.3%
23-25 years	33.7%	34.0%	32.3%	33.8%	31.8%	34.3%
26-28 years	35.6%	32.2%	32.2%	39.0%	30.4%	30.6%
29-35 years	30.3%	32.7%	37.0%	38.8%	31.9%	29.3%
Physical exercise (frequency)						
Never	59.5%	27.2%	13.3%	62.5%	24.4%	13.1%
Less than once a week	47.1%	34.2%	18.8%	51.6%	28.2%	20.1%
Once a week	39.9%	35.3%	24.8%	40.2%	35.7%	24.0%
2–3 times per week	31.1%	35.5%	33.5%	31.6%	34.5%	34.0%
Almost every day	24.6%	31.4%	44.1%	22.0%	31.3%	46.8%
Physical exercise (intensity)						
I take it easy without breaking into a sweat or losing my breath	43.5%	34.6%	21.9%	48.0%	29.7%	22.3%
I push myself so hard that I lose my breath and break into a sweat	31.0%	34.7%	34.3%	31.4%	34.1%	34.5%
I push myself to near-exhaustion	27.0%	31.0%	42.1%	27.4%	30.4%	42.2%
Physical exercise (duration)						
Less than 15 minutes	55.0%	29.0%	16.0%	58.5%	25.4%	16.2%
15–29 minutes	43.5%	34.8%	21.7%	42.8%	31.7%	25.5%
30 minutes to 1 hour	33.4%	34.3%	32.3%	35.5%	33.5%	30.9%
More than 1 hour	27.9%	34.5%	37.6%	28.2%	32.8%	39.0%
Top athlete						
Yes	18.8%	26.9%	54.3%	15.6%	23.0%	61.3%
No	24.9%	31.6%	43.5%	22.2%	31.8%	46.0%

*Note.* All *p* < .001. *p* values refer to the results from the chi-squared tests.

**Figure 2:**
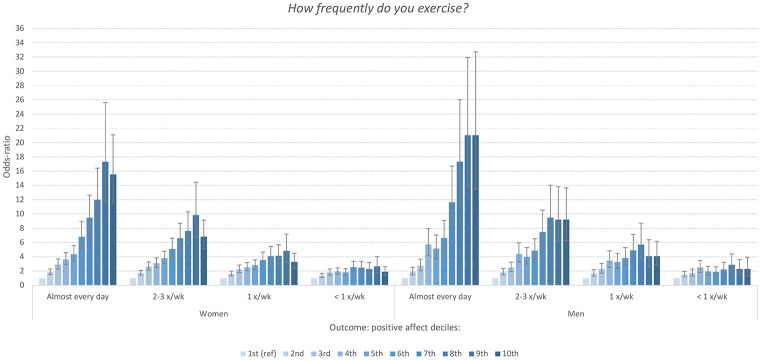
Odd-Ratios of Frequency of Physical Exercise Associated With Deciles of the Positive Affect (PA) Subscale of PANAS Stratified by Sex in the SHoT2018 Study *Note.* Reference category: *never*. Error bars represent 95% confidence intervals.

Figure 2 displays the results from the multinomial regression analysis examining the predictive effect of each physical exercise item on the level of PA, operationalized by deciles on the PA-subscale. As shown, there was a strong dose-response relationship between frequency of physical exercise and PA level. Although the associations were significant across all response categories of physical exercise (compared to “never”), the odds-ratios were especially strong among students reporting to train multiple times per week. Similarly, the effect sizes gradually increased parallel to elevating PA deciles. For example, students training every day had more than 20-fold increased odds of having a PA-score above the 90^th^ percentile (compared to the lowest decile). The correlations between the total PA score and exercise frequency for men and women were *r* = 0.28 and *r* = 0.23, respectively (both *p*s < .001).

**Figure 3:**
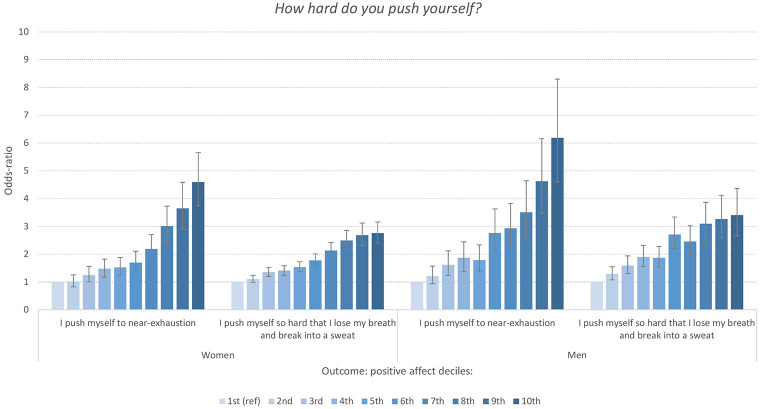
Odd-Ratios of Intensity of Physical Exercise Associated With Deciles of the Positive Affect Subscale of PANAS Stratified by Sex *Note.* Reference category*: I take it easy without breaking into a sweat or losing my breath*. Error bars represent 95% confidence intervals.

**Figure 4:**
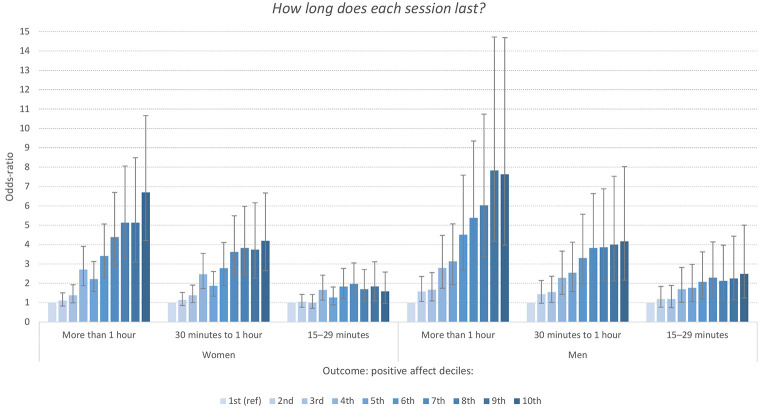
Odd-Ratios of Duration of Physical Exercise Associated With Deciles of the Positive Affect Subscale of PANAS Stratified by Sex *Note.* Reference category*: “Less than 15 minutes”*. Error bars represent 95% confidence intervals.

A similar pattern was observed for the item assessing the *intensity and duration* of physical exercise and PA level. As displayed in Figure 3, the harder the exercise, the higher the odds-ratio between physical exercise and PA. Similar to the frequency item, there was a clear dose-response association for both men and women. The *duration* of the physical exercise (Figure 4) was also associated with PA level in a similar manner: the *longer* the exercise, the stronger the association with high levels of PA. For example, both men and women reporting an average duration of exercise of more than one hour had between six to eight times increased odds of scoring in the top decile of the PA-subscale.

### What Is the Relationship Between Exercise and Positive Affect in Top Athletes?

**Figure 5:**
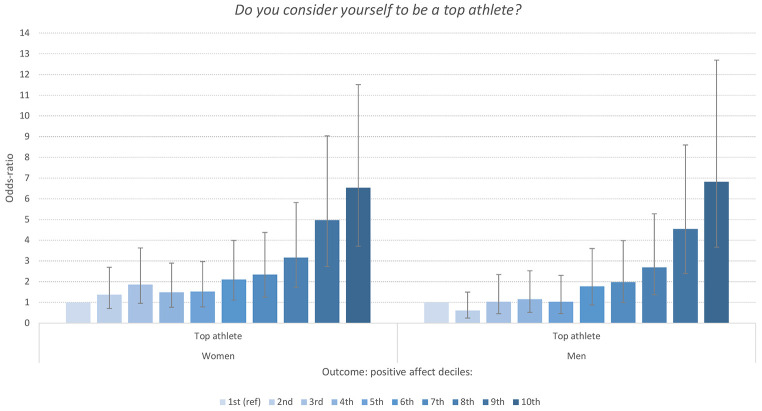
Odd-Ratios of Being a Top Athlete Associated With Deciles of the Positive Affect Subscale of PANAS Stratified by Sex *Note.* Reference group: Those not indicating that they are a top athlete. Error bars represent 95% confidence intervals.

Finally, students considering themselves to be a top athlete had significantly higher odds of also having a high level of PA. As shown in Figure 5, the associations were also here in a dose-response manner, although the associations were particularly strong for the top two deciles of the PA-subscale (above the 80^th^ percentile). These patterns were similar for both men and women, and there were no significant sex interactions for any of the analysis.

### Is This Graded Association True in Both Lean and Overweight/Obese Participants?

**Figure 6:**
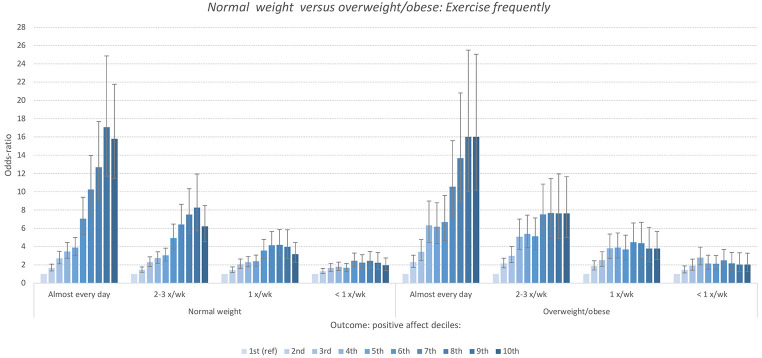
The Association Between Exercise Frequency and Decile of PANAS Positive Affect for Normal and Overweight/Obese Participants

As shown in Figure 6, across healthy weight and obese/overweight categories, the association persists and is seen to be nearly identical across the two BMI groups at different levels of exercise.

### What Happens When the Word Active Is Separated From the PANAS?

Removing the adjective “active” from the PANAS cut the association between PA and exercise frequency a great deal. As shown in Figure 7, when considering exercise frequency, while the graded association remained robust and in the same pattern, some associations dropped by 50%. For example, the odds for daily exercisers of being in the highest PA decile went from approximately 18x to 9x (Figure 7, Panel 1). The degree of change in odds was less severe at lower levels of exercise. While not as dramatic a change, but in the same direction, the odds of being in the top decile for those who push themselves the hardest during exercise dropped from a 4.8 to a 3.8 (Figure 7, Panel 2). On the flip side this pattern was present in other activity outcomes, although not to the same degree. For example, in the duration of exercise outcome, there was no observable change from removing “active” from the 15-29 minute of exercise at a time subset (Figure 7, Panel 3).

**Figure 7:**
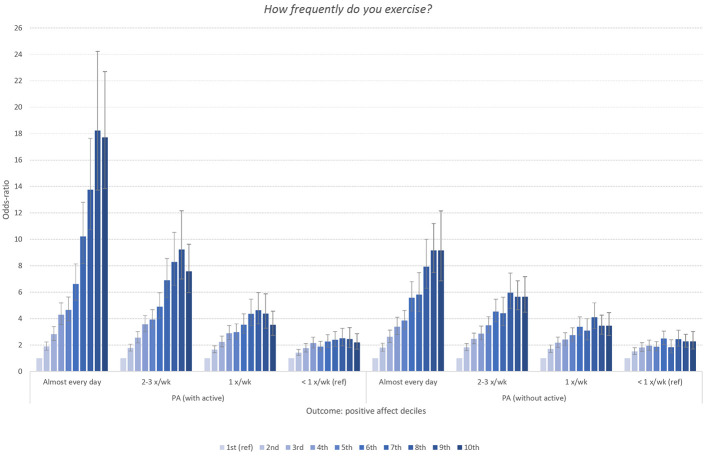
Panel 1

**Figure 7:**
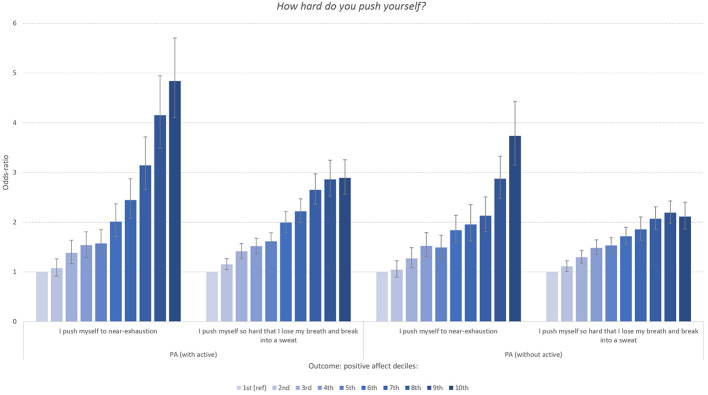
Panel 2

**Figure 7:**
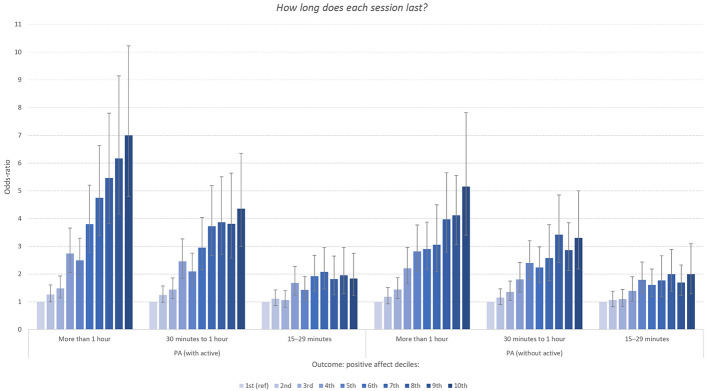
Panel 3 *Note.* (Panels 1, 2, 3). Odd-ratios of frequency, intensity and duration of physical exercise (versus lowest categories displayed in Figure 2, Figure 3, and Figure 4) associated with deciles of the positive affect (PA) subscale of PANAS organized by full scale (left) and the PANAS minus the word “active” (right). Error bars represent 95% confidence intervals.

## Discussion

Overall, this study replicates past findings indicating a strong association between PA and exercise in a large general population study of over 50,000 Norwegian young adults, but it also adds a great deal of new information. First, we show for the first time a surprisingly clear and strong dose-response relationship between PA and physical exercise across *all* three self-reported assessments (i.e., duration, frequency, intensity). The magnitude and slope of the dose-response relationships were particularly driven by those participants who exercise regularly. For example, those training every day had a more than *20-fold increased odds* of being in the top 10% of PA scores (versus those not exercising regularly), albeit with large confidence intervals. Similarly, when indexing other measures of self-reported exercise, those reporting more than 1 hour per session had between six to eight times increased odds of scoring in the top decile of the PA-subscale. Also, those who considered themselves to be elite athletes were overrepresented in the top 80th percentile of PA with approximately five to six times the odds of being in these top groups versus non elite athletes. Importantly, these effects were seen across all levels of body mass index indicating that this is not simply about physical fitness, but perhaps more about *actual* activity. Relevant to this interpretation, removing the word active from the PANAS made a large difference in the size of the PA association with exercise, in some cases, cutting the linkage size by 50% in the highest and most consistent exercisers, but having less of a dramatic effect in the less active individuals.

All together, these results indicate that high PA, as assessed by the PANAS, is in fact picking up on activity to a large extent, especially when assessing regular exercisers. That is, the majority of the *most* positive people are regular exercisers, and in some cases, elite athletes. The robust effect of removing the activity item from the PANAS highlights further this issue, that is, that the highest people in PA are the most active people, partially because, the PANAS *measures* activity. People who say they are *feeling* active, are by in large *actually* more active. Clearly qualitative work is needed to explore what people are evaluating in their lives and emotions when they answer these PANAS PA items, as well as work tying objective fitness (e.g., as measured by V02max) to PANAS active and other PA items.

We must also ask the more critical question of how these results impacts our interpretation of the literature connecting PA to better health? The findings clearly cast some doubt on health studies utilizing the PANAS PA or similarly active measures of PA (e.g., vigor), especially among results that don’t account for the effects of physical activity in some way. That said, even if they did, given the typically limited fitness measures used in some studies, more work is probably needed to ensure that it is not simply the most physically fit, active, and healthy people driving these findings or some other related third variable (e.g., cardiovascular health, mitochondrial function) (Fuchs, 2015; Picard, 2011; Stevens, 2009). The study also raises questions about whether PA intervention studies in ill populations are in fact increasing the correct factors for health promotion since much of this work is based on past found associations between active PA and health. The current study points to the possibility that exercise may be a more important or sufficient target in some populations (a popular intervention in some diseased or high risk populations) (Ornish et al., 1998; Ryan, Cassidy, Noorduyn, & O’Connell, 2017; Theou et al., 2011; e.g., van der Wardt et al., 2020) as opposed to focusing on emotion in interventions.

In future research exploring the relationship between PA and health we recommend researchers consider taking extra effort to separate the effects of PA from physical activity when exploring health outcomes. This might be done by utilizing objective fitness indicators such as VO2max, accelerometers, extensive exercise and activity self-reports, in addition to covarying perceived health which is likely to relate strongly to fitness. It is only with these deeper and more objective approaches that we will begin to understand when feelings of positivity are promoting health versus activity levels (i.e., healthiness) promoting health.

This study has both strengths and weaknesses. While it is well powered and has an array of physical exercise assessments, it is limited by its cross-sectional design and reliance on self-report. Generalization is also limited to young, healthy, and primarily Caucasian samples. Given the high self-reported exercise levels of this sample, it would be interesting to also contrast these levels against objective activity assessments as well as to look at less active and older samples.

In addition, the use of a state (current) affect scale was weaker than that of a trait (long lasting) affect scale, although the two are known to be highly correlated (Diener & Emmons, 1984). It should also be noted that some of the 95% confidence intervals were quite large, especially for the top deciles of the PA scale. This should be kept in mind when interpreting the results. Finally, we could have opted to remove other active/high arousal affect items, such as the word “strong”, from the PANAS to examine the resulting change in association with exercise. We chose “active” due to its more regular use in affect and health assessments as well as due to past results revealing that it was clearly the most tied to health (specifically, all-cause mortality with a hazard ratio of ~1.9). The association of the word “strong”, for example, was comparable to many other PA items (HR ~ 1.4 which was similar to PANAS adjectives like interested and attentive) (Petrie et al., 2018).

Overall, this study shows strongly that exercise and positive emotions are closely intertwined, especially for the healthiest and most fit individuals. Future work should examine how the same effects are found with objective measures of activity and fitness, and should also further examine the implications for physical health outcomes. That is, when examining PA and health connections, to what extent do these change if we focus on PA measures that do not tap energy, felt vigor, and activity? What happens when we take great efforts to account for activity and fitness? From this data, we might infer that this would not have major implications for sedentary samples, however, for samples that include active individuals, effects may change drastically. It is essential that those of us interested in PA and health start measuring exercise well and that we take the possible different interpretations of high activity/arousal PA effects into account. The extent that we discover that activity levels underlie a large amount of previously observed PA health benefits, it may be the case that activity interventions (with or without PA) may be a more fruitful approach to improving health.

## Author Note

Twitter Handles for authors: @sarahpressman @KeithPetrie @borgesivertsen
